# Drain Current Stress-Induced Instability in Amorphous InGaZnO Thin-Film Transistors with Different Active Layer Thicknesses

**DOI:** 10.3390/ma11040559

**Published:** 2018-04-05

**Authors:** Dapeng Wang, Wenjing Zhao, Hua Li, Mamoru Furuta

**Affiliations:** 1 Key Laboratory of Applied Surface and Colloid Chemistry, Ministry of Education, Shaanxi Key Laboratory for Advanced Energy Devices, Shaanxi Engineering Lab for Advanced Energy Technology, School of Materials Science and Engineering, Shaanxi Normal University, Xi’an 710119, China; wenjingz@snnu.edu.cn (W.Z.); lihua0405@snnu.edu.cn (H.L.); 2 School of Environmental Science and Engineering, Kochi University of Technology, Kami, Kochi 782-8502, Japan; 3 Center for Nanotechnology in Research Institute, Kochi University of Technology, Kami, Kochi 782-8502, Japan

**Keywords:** drain current stress, instability, InGaZnO, thin-film transistors, active layer thickness

## Abstract

In this study, the initial electrical properties, positive gate bias stress (PBS), and drain current stress (DCS)-induced instabilities of amorphous indium gallium zinc oxide (a-IGZO) thin-film transistors (TFTs) with various active layer thicknesses (*T*_IGZO_) are investigated. As the *T*_IGZO_ increased, the turn-on voltage (*V*_on_) decreased, while the subthreshold swing slightly increased. Furthermore, the mobility of over 13 cm^2^·V^−1^·s^−1^ and the negligible hysteresis of ~0.5 V are obtained in all of the a-IGZO TFTs, regardless of the *T*_IGZO_. The PBS results exhibit that the *V*_on_ shift is aggravated as the *T*_IGZO_ decreases. In addition, the DCS-induced instability in the a-IGZO TFTs with various *T*_IGZO_ values is revealed using current–voltage and capacitance–voltage (*C*–*V*) measurements. An anomalous hump phenomenon is only observed in the off state of the gate-to-source (*C*_gs_) curve for all of the a-IGZO TFTs. This is due to the impact ionization that occurs near the drain side of the channel and the generated holes that flow towards the source side along the back-channel interface under the lateral electric field, which cause a lowered potential barrier near the source side. As the *T*_IGZO_ value increased, the hump in the off state of the *C*_gs_ curve was gradually weakened.

## 1. Introduction

Recently, amorphous indium gallium zinc oxide (a-IGZO), as a representative of an amorphous metal oxide-based semiconductor, has been widely investigated for use in the active layer of thin-film transistors (TFTs) due to its high electron mobility, good transparency in visible light, chemical and thermal stability, low temperature processing, and smooth surface [1,2,3,4]. The a-IGZO TFT with excellent electrical properties, such as high mobility (*μ*) of over 10 cm^2^·V^−1^·s^−1^ and low values of subthreshold swing, has become one of the research hotspots for the advanced display application in next-generation active-matrix liquid crystal displays (AM-LCDs) and active-matrix organic light-emitting diodes (AM-OLEDs) [5,6,7,8]. Hitherto, AM-OLEDs driven by the a-IGZO TFTs involve two or three transistors and one capacitor current-biased voltage-programmed pixel circuit. Therefore, the stability of the a-IGZO TFTs under long-term current-bias is a critical issue for these circuits in AM-OLEDs. However, the a-IGZO TFTs inevitably suffer gate and drain bias stresses during practical operation conditions, leading to device instability and hindering their development for commercial products [9,10]. Fujii et al. [11] have investigated the increase in internal temperature of the IGZO TFTs when the device was operated in the saturation region. Choi et al. [12] have reported that the electron-hole pair generation by impact ionization near the drain side contributed to the negative shift of the threshold voltage of IGZO TFTs with wide channel width under a high gate and drain bias stress. Valdinoci et al. [13] have reported that the electron-hole pair generation by impact ionization near the drain region caused the floating body effect in high *μ* poly-Si TFTs. Consequently, the electrical stability under drain current stress was considered to be an important issue, especially for high-*μ* oxide TFTs.

Moreover, the active layer thickness is an important parameter to adjust device electrical properties, such as on/off ratio, threshold voltage, and field effect mobility [14,15,16]. As reported in previous publications, the device performance is significantly influenced by the semiconductor/gate insulator (GI) interfacial density [17,18] and the active layer trap density [19], indicating that the total trap density increases with the increase in the active layer thickness [20], which can effectively modify the threshold voltage and field effect mobility. Therefore, the impact of the active layer thickness (*T*_IGZO_) on the instability induced by the positive gate bias stress (PBS) and the drain current stress (DCS) in a-IGZO TFTs should be well investigated.

In this study, the initial electrical properties and PBS and DCS-induced instabilities of a-IGZO TFTs with various *T*_IGZO_ are investigated. Moreover, the DCS-induced instability in the a-IGZO TFTs with various *T*_IGZO_ is revealed by the combination of current-voltage (*I*–*V*) and capacitance-voltage (*C*–*V*) measurements.

## 2. Experimental

**Figure 1:**
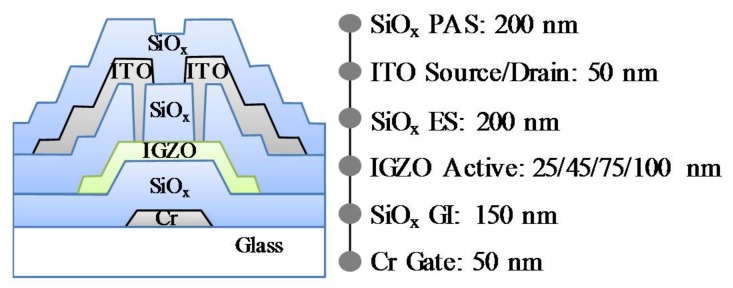
A schematic cross-sectional view and fabrication process of the bottom-gate indium gallium zinc oxide (IGZO) thin-film transistor (TFT).

A schematic cross-sectional view and fabrication process of the bottom-gate IGZO TFT are shown in Figure 1. The fabrication procedure for the a-IGZO TFT is as follows. A chromium (Cr) gate electrode is firstly formed on a glass substrate. A SiO_x_ gate insulator (GI) with a thickness of 150 nm is then deposited at 350 °C by plasma-enhanced chemical vapor deposition (PECVD). The a-IGZO layer with thicknesses of 25 nm, 45 nm, 75 nm, and 100 nm are deposited at 160 °C from a sintered IGZO ceramic target by direct current (DC) magnetron sputtering with a mixed gas of Ar/O_2_ = 29.4/0.6 sccm at a deposition pressure of 1 Pa. After patterning the IGZO film as an active channel, a SiO_x_ film (200 nm) as an etch stopper is deposited by PECVD. Source and drain electrodes are formed using indium tin oxide (ITO) via contact holes. A 200-nm thick SiO_x_ passivation layer is also deposited by PECVD. Finally, the IGZO TFTs are annealed in N_2_ ambient at 350 °C for 1 h before electrical measurements. The channel width (*W*) and length (*L*) the IGZO TFTs are 50 μm and 20 μm, respectively. All of the *I*–*V* characteristics are measured using an Agilent 4156C precision semiconductor parameter analyzer. The *C*–*V* measurements, the channel capacitance (*C*_gc_), the gate-to-source capacitance (*C*_gs_), and the gate-to-drain capacitance (*C*_gd_), are measured at 1 kHz and an alternating current (AC) level of 100 mV. All of the measurements are carried out at room temperature in ambient air.

## 3. Results and Discussion

**Figure 2:**
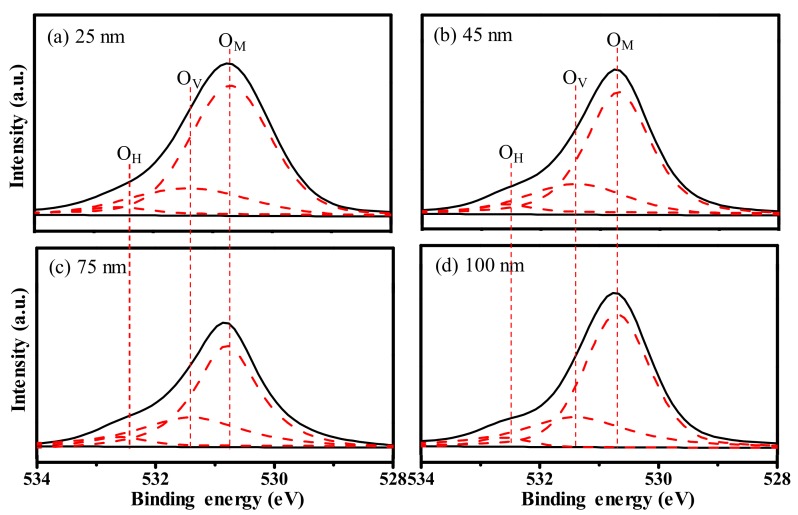
X-ray photoelectron spectroscopy (XPS) spectra of O 1s peaks of the IGZO films with the thickness of (**a**) 25 nm; (**b**) 45 nm; (**c**) 75 nm; and (**d**) 100 nm, respectively.

To investigate the thickness impact on the chemical properties and bonding states of the IGZO films, an X-ray photoelectron spectroscopy (XPS, ESCALAB250Xi, Thermo Fisher Scientific, Waltham, MA, USA) measurement is performed. Figure 2 shows the O 1s XPS spectra of the IGZO films with various thicknesses. The O 1s spectra can be resolved into three nearly Gaussian distribution peaks approximately centered at 530.7 eV, 531.4 eV, and 532.6 eV. The peaks at the binding energy of 530.7 eV (labeled as O_M_), 531.4 eV (labeled as O_V_), and 532.6 eV (labeled as O_H_) are attributed to the O^2−^ ions combined with the metal atoms, oxygen deficiency, and hydroxyl groups in a stoichiometric IGZO structure, respectively [21]. The positions, areas, and area ratios of the O 1s three peaks for the IGZO films with various thicknesses are summarized in Table 1. For the 25-nm thick IGZO film, the O_M_/(O_M_ + O_V_ + O_H_) and O_V_/(O_M_ + O_V_ + O_H_) area ratios are 74.4% and 22.4%, respectively. It is suggested that the IGZO film contains a small quantity of oxygen-related defects during the short-time fabrication in the chamber. With the increase in the deposition duration, the O_V_/(O_M_ + O_V_ + O_H_) area ratio evidently increased, whereas the O_M_/(O_M_ + O_V_ + O_H_) area ratio obviously decreased. When the IGZO thickness is increased to 100 nm, the area ratios of O_V_/(O_M_ + O_V_ + O_H_) and O_M_/(O_M_ + O_V_ + O_H_) are changed remarkably to 29.1% and 67.7%, respectively. These results reveal that the long-time sputtering process deteriorates the quality of the IGZO film and accelerates the generation of the oxygen-related defects, which cause the unbalanced chemical bonds of metal and oxygen atoms. Interestingly, the area ratio of O_H_/(O_M_ + O_V_ + O_H_) keeps the small constant of ~3.2%, even for IGZO films fabricated under different durations, implying that the magnetron sputtering method is a promising approach to obtain a metal oxide semiconductor with small amounts of hydroxyl groups.

**Figure 3:**
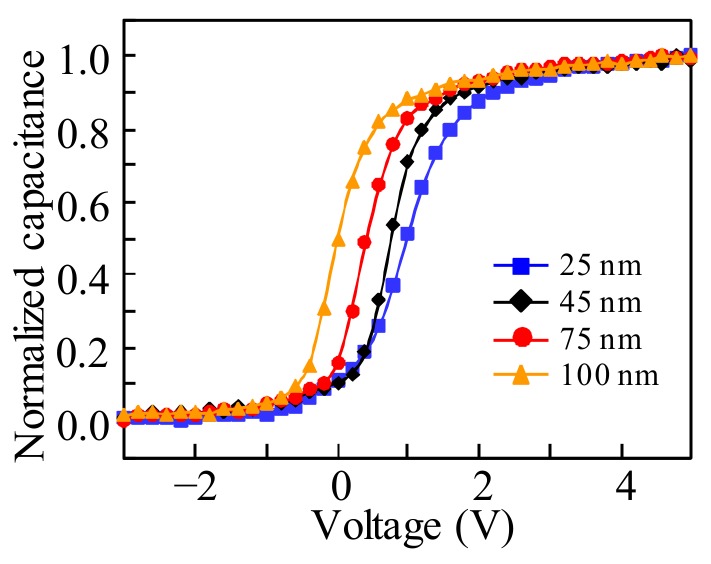
Capacitance–voltage (*C*–*V*) characteristics of the indium tin oxide (ITO)/IGZO/SiO_2_/Cr stack structure with various *T*_IGZO_.

Figure 3 illustrates the *C*–*V* plot as a function of the thickness of IGZO in the ITO/IGZO/SiO_2_/Cr stack structure. It is noted that the increase in the IGZO thickness induces a negative shift of the flat band voltage (*V*_FB_). The variation of the *V*_FB_ in the negative direction implies that the threshold voltage (*V*_th_) of the ITO/IGZO/SiO_2_/Cr stack structure-based TFTs can be adjusted by using the IGZO layer with various thicknesses. In addition, the maximum negative shift of *V*_FB_ is observed for the IGZO film with the thickness of 100 nm, which contributes to the largest negative shift of the *V*_th_.

**Figure 4:**
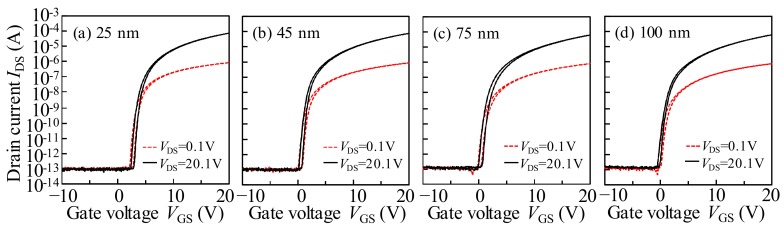
The initial transfer characteristics (*I*_DS_–*V*_GS_) of amorphous indium gallium zinc oxide (a-IGZO) TFTs with various *T*_IGZO_ of (**a**) 25 nm; (**b**) 45 nm; (**c**) 75 nm; and (**d**) 100 nm measured at *V*_DS_ of 0.1 and 20.1 V, respectively.

Figure 4 shows the initial transfer characteristics (*I*_DS_–*V*_GS_) of a-IGZO TFTs with various active layer thicknesses (*T*_IGZO_) measured at *V*_DS_ values of 0.1 V and 20.1 V. The electrical properties, such as field effect mobility in the saturation region (*μ*_sat_), *V*_on_ (defined by *V*_GS_ at *I*_DS_ of 1 nA), subthreshold swing (SS = d*V*_GS_/dlog_10_(*I*_DS_)), and hysteresis of the transfer curves (defined by the difference of *V*_GS_ at *I*_DS_ of 1 nA between the forward and reverse sweeps) are summarized in Table 2.

The saturation mobility *μ*_sat_ is calculated by fitting a straight line to the plot of the square root of *I*_DS_ versus *V*_GS_ based on the following equation [22]:

(1) $$I_{\mathrm{DS}}=\frac{\mu_{\mathrm{sat}}WC_{{\mathrm{SiO}}_{x}}}{2L}{\left(V_{\mathrm{GS}}-V_{\mathrm{th}}\right)}^{2}$$

where *W* and *L* are the channel width and length, respectively, and *C*_SiOx_ is the capacitance per unit area of the GI. When the *T*_IGZO_ is increased from 25 nm to 100 nm, the *μ*_sat_ is slightly degraded from 14.17 cm^2^·V^−1^·s^−1^ to 13.04 cm^2^·V^−1^·s^−1^. The *μ*_sat_ is affected by the quality of the active layer and the a-IGZO/GI interface. To confirm the influence of the *T*_IGZO_ on the quality of the a-IGZO/GI interface, the hysteresis behaviors of the IGZO TFT with various *T*_IGZO_ are extracted, as listed in Table 1. The identically negligible clockwise hysteresis is obtained regardless of the *T*_IGZO_, indicating that the good quality of the IGZO/GI interface is well kept during the fabrication processes for all of the IGZO TFTs. Moreover, the *V*_on_ and SS values are significantly changed from 2.32 V and 323 mV/dec. in the 25-nm thick IGZO TFT to −0.33 V and 475 mV/dec. in the 100-nm thick IGZO TFT, respectively. The degraded SS value and the shifted *V*_on_ in the negative *V*_GS_ direction can be commonly interpreted as consequences of the total defect states and free carrier numbers being increased as the *T*_IGZO_ values increased, which is consistent with previous publications [23,24] and in agreement with the *C*–*V* measurements in Figure 3. Generally, the SS value is an indicator of the maximum area density of state (*N*_t_), including the interfacial (*D*_it_) and the semiconductor bulk traps (*N*_bulk_). The *N*_t_ value can be extracted from following equation [25]:

(2) $$N_{t}=\left(\frac{SS\times \log\left(e\right)}{kT/q}-1\right)\frac{C_{{\mathrm{SiO}}_{x}}}{q}$$

where *q* is the electron charge, and *k* is the Boltzmann constant. The *N*_t_ values were 6.53 × 10^11^, 7.62 × 10^11^, 8.83 × 10^11^, and 1.03 × 10^12^ cm^–2^·eV^–1^ for the IGZO TFTs with the *T*_IGZO_ values of 25 nm, 45 nm, 75 nm, and 100 nm, respectively. Obviously, the *N*_t_ is increased with the increase in the *T*_IGZO_ value, which is consistent with the XPS results. The results exhibit that the change in the *N*_t_ mainly originated from the *N*_bulk_, owing to the similar a-IGZO/GI interfacial quality.

**Figure 5:**
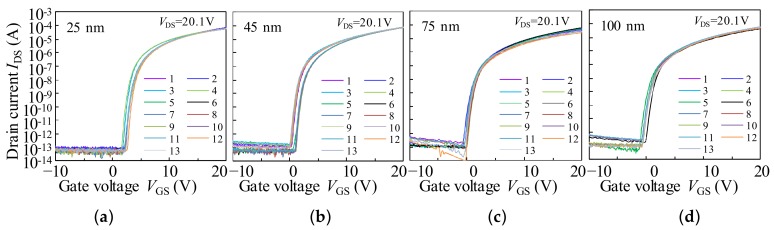
The *I*_DS_–*V*_GS_ of 13 individual TFTs with various *T*_IGZO_ of (**a**) 25 nm; (**b**) 45 nm; (**c**) 75 nm; and (**d**) 100 nm measured at *V*_DS_ of 20.1, respectively.

To confirm the uniformity and reproducibility of the a-IGZO TFTs with various *T*_IGZO_, the *I*_DS_–*V*_GS_ curves of the 13 individual devices measured at *V*_DS_ of 20.1 V are shown in Figure 5, respectively. The corresponding electrical properties, such as *μ*_sat_, *V*_on_, SS, and hysteresis, are listed in Table 3. Notably, the statistical distribution of all of the parameters has the same tendency as described in Table 1 and small standard deviations, thereby indicating very good reproducibility in the fabricated a-IGZO TFTs.

**Figure 6:**
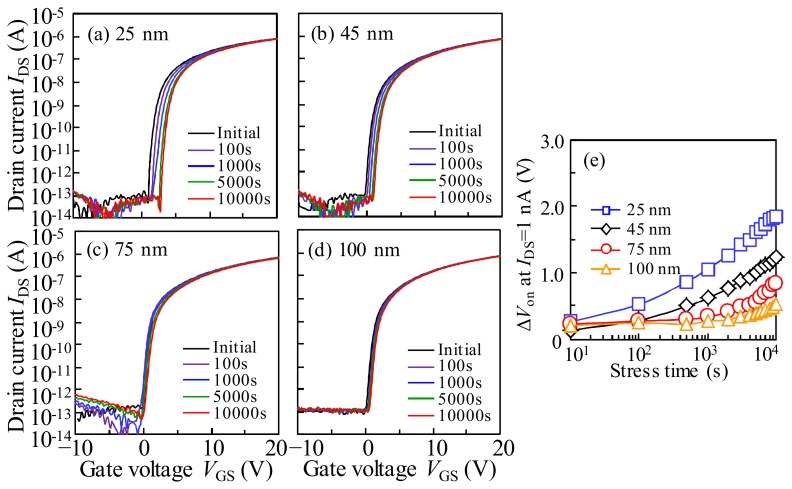
Evolution of transfer characteristics in the a-IGZO TFT with the *T*_IGZO_ values of (**a**) 25 nm; (**b**) 45 nm; (**c**) 75 nm; and (**d**) 100 nm as a function of the 20 V positive gate bias stress (PBS) duration for 10^4^ s; and (**e**) the variation in Δ*V*_on_ as a function of PBS duration for the a-IGZO TFTs with various *T*_IGZO_.

To investigate the impact of the *T*_IGZO_ on the stability of a-IGZO TFTs, the positive bias stress (PBS) is carried out. Figure 6a–d shows the variation in the transfer characteristics of the a-IGZO with various *T*_IGZO_ under PBS with a *V*_GS_ value of 20 V. The variation in *V*_on_ (Δ*V*_on_) as a function of PBS duration for the a-IGZO TFTs with various *T*_IGZO_ values is shown in Figure 5e. It is found that the transfer characteristics for all of the TFTs under PBS shift parallel in the positive *V*_GS_ direction without SS degradation, indicating that the electrons are trapped at the interface of the a-IGZO or in the GI without introducing any defects. When the *T*_IGZO_ is decreased from 100 nm to 25 nm, the Δ*V*_on_ is remarkably increased from 0.52 V to 1.85 V after the stress duration of 10^4^ s. The obtained results can be explained by the vertical electrical field distribution. Generally, the electric potential exponentially declines inside the active layer, and has a maximum transfer length called the Debye length. For the a-IGZO TFT, the calculated Debye length was ~40 nm [19]. When the *T*_IGZO_ is less than Debye transfer length (*T*_IGZO_ = 25 nm), the surface potential will exponentially decline into the whole active layer. Therefore, with the decrease in the *T*_IGZO_ value, the electrical field will be enhanced. Under PBS, the electrons in the thinner *T*_IGZO_ will be accelerated by the enhanced surface field, which are accumulated by electrical field energy and are trapped at the interface of the a-IGZO/GI or in the GI under the positive bias, leading to the large positive *V*_GS_ shift. When the *T*_IGZO_ increased to more than the Debye length of 40 nm, the electric field at the front-interface becomes lower, contributing to the few electrons that are trapped at the front-interface, which exhibits the small Δ*V*_on_ with the increase in the *T*_IGZO_ value.

**Figure 7:**
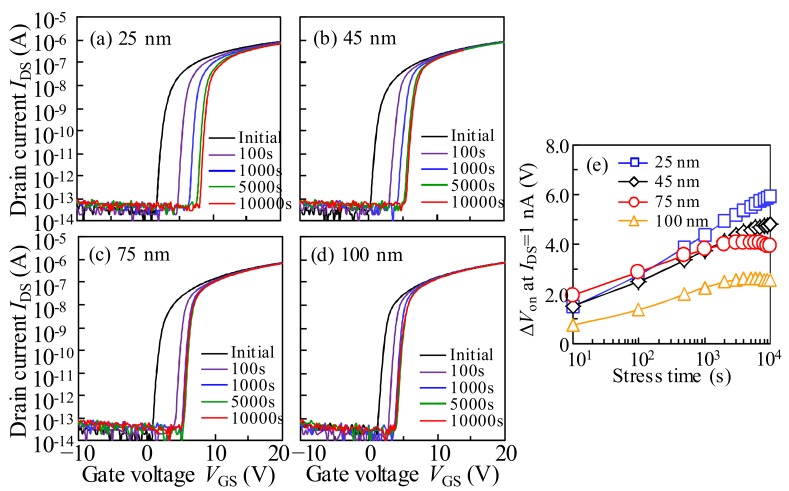
Evolution of transfer characteristics in the a-IGZO TFT with the *T*_IGZO_ values of (**a**) 25 nm; (**b**) 45 nm; (**c**) 75 nm; and (**d**) 100 nm as a function of the drain current stress (DCS) (*V*_GS_ = *V*_DS_ = 25 V) duration for 10^4^ s, and (**e**) the variation in Δ*V*_on_ as a function of DCS duration for the a-IGZO TFTs with various *T*_IGZO_.

To simulate the practical operation conditions, the drain current stress (DCS) is applied to the a-IGZO TFTs with various *T*_IGZO_ values. Figure 7a–d shows the variation in the transfer characteristics of the a-IGZO with various *T*_IGZO_ under DCS with *V*_GS_ = *V*_DS_ = 25 V. The variations in Δ*V*_on_ as a function of the DCS duration for the a-IGZO TFTs with various *T*_IGZO_ values are shown in Figure 7e. Noticeably, the transfer curves of all of the a-IGZO TFTs exhibit a parallel shift in the positive *V*_GS_ direction without SS degradation during the DCS duration. In the initial stage of DCS (<100 s), all of the transfer curves shift significantly towards the *V*_GS_ direction without SS degradation. In the subsequent stage (>100 s), the amplitude of Δ*V*_on_ becomes weakened, and the Δ*V*_on_ increases with the decrease in the *T*_IGZO_ after the DCS for 10^4^ s.

**Figure 8:**
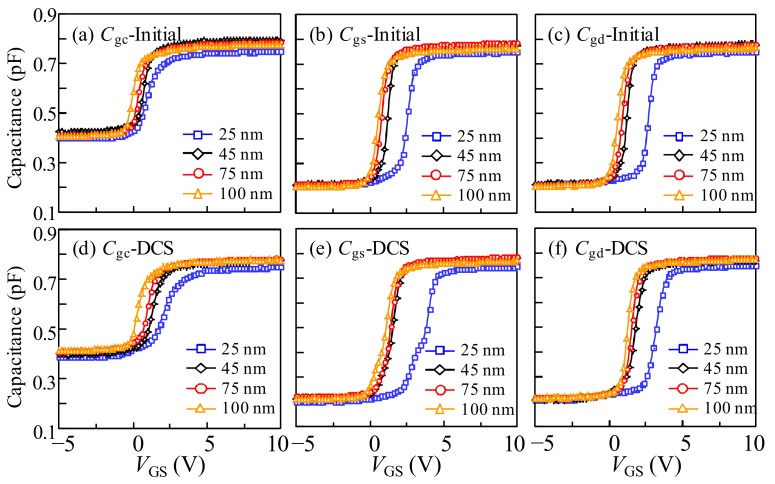
*C*–*V* curves of (**a**) *C*_gc_; (**b**) *C*_gs_; and (**c**) *C*_gd_ in the initial and (**d**) *C*_gc_; (**e**) *C*_gs_; and (**f**) *C*_gd_ after a DCS duration of 10^4^ s with various *T*_IGZO_.

To clarify the mechanism of the DCS-induced instability in the a-IGZO TFTs with various *T*_IGZO_, the *C*–*V* analyses of *C*_gc_, *C*_gs_, and *C*_gd_ before and after DCS duration of 10^4^ s are carried out, as shown in Figure 8. Compared with the *C*_gc_ curves of the a-IGZO TFTs with various *T*_IGZO_ values in the initial stage and after DCS duration, all of the *C*_gc_ curves exhibited a positive *V*_GS_ shift with distortion in the off state of the *C*–*V* curves. The shift of the *C*–*V* curves is weakened as the *T*_IGZO_ value increases, which has a similar tendency to the *I*–*V* curves, as shown in Figure 7. However, the shift amplitude of the *C*–*V* curves is smaller than that of the *I*–*V* curves, indicating that the less free carriers are trapped, or the trapped carriers are partly de-trapped during the *C*–*V* measurement after the DCS. Furthermore, the hump phenomenon in the off state of the *C*–*V* curves becomes weakened as the *T*_IGZO_ values increase, which is hardly observed in the *I*–*V* curves. To further investigate the origin of the hump phenomenon in the off state of the *C*–*V* curves, the *C*_gs_ and *C*_gd_ values before and after the DCS are measured. Note that the both *C*_gs_ and *C*_gd_ curves exhibit a parallel shift in the positive *V*_GS_ direction. However, the hump phenomenon is only observed in the *C*_gs_ curve rather than the *C*_gd_ curve during the DCS.

**Figure 9:**
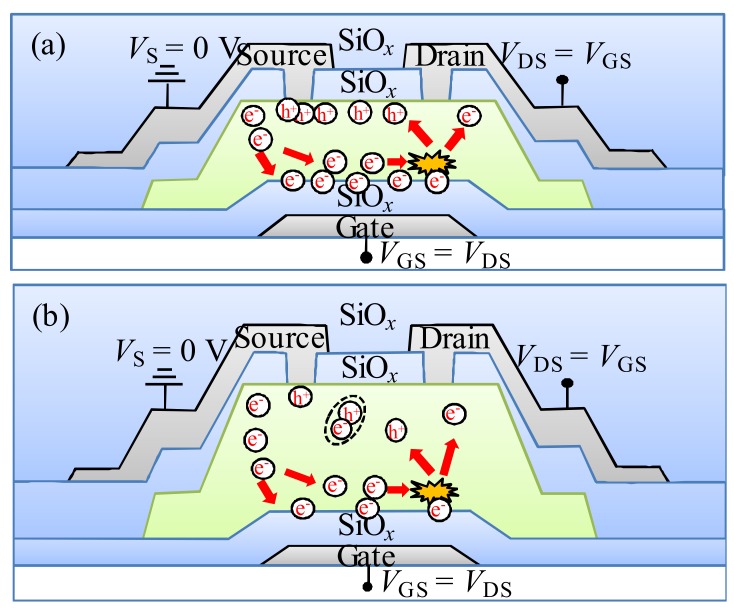
The schematic diagram of the mechanism of DCS-induced instability in the IGZO TFT with the *T*_IGZO_ value of (**a**) 25 nm and (**b**) 45 nm, 75 nm, or 100 nm.

In terms of the a-IGZO TFT with a *T*_IGZO_ of 25 nm under the DCS (*V*_DS_ = *V*_GS_), the electrons are transported from the source to drain side along the front-channel interface, which contributes to a depletion region near the drain side. Combined with the case of 25-nm thick IGZO TFT under the PBS, in the initial stage of DCS (<100 s), the electrons are accelerated to the front-channel under the high vertical electric field. Then, they are trapped at the interface of the a-IGZO/GI or injected into the GI, resulting in a significantly positive *V*_GS_ shift of the transfer curve. Simultaneously, the electrons are accelerated from the source to the drain side under the lateral electric field, resulting in the impact ionization occurring at the drain side of the channel [12]. Subsequently, the electron-hole pairs are generated by impact ionization near the drain side. The generated electrons and holes are collected at the front-channel and the etch-stopper/IGZO (back-channel) interfaces, respectively. The generated holes flow towards the source side along the back-channel interface and cause a lowered potential barrier near the source side, leading to the additional charge response in the *C*–*V* measurement, which contributes to the hump in the off state of the *C*_gs_ curve. The schematic diagram of DCS-induced degradation in the IGZO TFT with the *T*_IGZO_ of 25 nm is illustrated in Figure 9a. In the subsequent stage (>100 s), with the extension of DCS duration, the more generated holes are accumulated near the source side, which contributes to the increase in the body potential. Therefore, the Δ*V*_on_ of the transfer curve is weakened with the DCS duration.

When the *T*_IGZO_ value is increased to 45 nm, a similar phenomenon is observed in the a-IGZO TFT under the DCS of 10^4^ s. Due to the reduction of the vertical electric field, the amount of the trapped electrons are decreased at the interface of the channel/GI or into the GI, leading to the smaller Δ*V*_on_ of the transfer curves compared with the 25-nm thick IGZO TFT, which is in agreement with the *I*–*V* and *C*–*V* results. Meanwhile, the impact ionization occurs near the drain side under the lateral electric field. The electrons are accelerated from the source to the drain side, which induces the generation of the electron-hole pair near the drain side. The generated holes drift towards the source side along the back-channel interface. Due to the amount of free electrons that increase with the increase in the *T*_IGZO_, the recombination probability of the holes and electrons are enlarged during the hole drifting. The number of the collected holes at the source side is reduced, contributing to the small hump in the off state of the *C*_gs_ curve. When the *T*_IGZO_ is further increased to 75 nm or 100 nm, the positive *V*_GS_ shift of the transfer curves is significantly decreased due to the weaker vertical electric field with the increase in the *T*_IGZO_ value, contributing to the slightly positive *V*_GS_ shift of the *I*–*V* and *C*–*V* curves. The schematic diagram of the mechanism of DCS-induced instability in the IGZO TFT with the thicker *T*_IGZO_ is illustrated in Figure 9b. The generated holes induced by the impact ionization in the drain region are drifted from the drain to the source side along the back-channel under the vertical and lateral electric fields. The holes would suffer easily from the recombination with the more free electrons in the thicker IGZO layer. Therefore, the slight hump in the off state of the *C*_gs_ curve is attributed to the few holes that are accumulated at the back-channel near the source side.

Besides the *T*_IGZO_ value, the architecture of devices also plays a critical role in the DCS-induced instability of the TFTs. On the basis of our previous publication [26], the role of impact ionization is strongly dependent on channel scale, and exhibits two types of dependences on channel length and width. When the DCS is applied to the TFTs with a fixed channel length and different channel widths, the stronger impact ionization can be observed for the wider channel width TFT, leading to the high heating temperature. On the other hand, when the DCS is carried out on the devices with a fixed channel width and different channel lengths, the stronger impact ionization can be obtained for the shorter channel length TFT. Therefore, besides the proper *T*_IGZO_, the a-IGZO TFTs with the relatively long length and short width may effectively minimize the impact ionization effect, improving the DCS-induced stability of the a-IGZO TFTs.

## 4. Conclusions

In this study, the initial electrical properties, PBS, and DCS-induced instabilities of a-IGZO TFTs with various *T*_IGZO_ are investigated. As the *T*_IGZO_ values increased, the *V*_on_ decreased, while the SS slightly increased because the total defect states and free carrier numbers were increased as the increase in the *T*_IGZO_. It is found that the Δ*V*_on_ under PBS is aggravated as the decrease in the *T*_IGZO_, which is due to the enhancement of the vertical electrical field in the channel. In addition, the DCS-induced instability in the a-IGZO TFTs with various *T*_IGZO_ values is revealed by the combination of *I*–*V* and *C*–*V* measurements. The *C*–*V* results indicate that an anomalous hump phenomenon is only observed in the off state of the *C*_gs_ curve for all of the a-IGZO TFTs. This is because the impact ionization occurs near the drain side of the channel and the generated holes flow towards the source side along the back-channel interface under the lateral electric field, which causes a lowered potential barrier near the source side. Since the amount of free electrons increase with the increase in the *T*_IGZO_ values, the recombination probability of the generated holes and electrons are enlarged during the hole drifting, leading to the weakened hump phenomenon as the the *T*_IGZO_ values increased. This study points out that material and fabrication engineering in the drain region should be well considered, even for the high-performance oxide TFTs.

## Figures and Tables

**Table 1:** The position, areas, and area ratios of the deconvoluted O 1s peaks for the IGZO films with various thicknesses.

O 1s		IGZO Thickness (nm)			
		25	45	75	100
O_M_	Position (eV)	530.7	530.7	530.7	530.7
	Area	177500	159500	158000	156000
O_V_	Position (eV)	531.4	531.4	531.4	531.4
	Area	53500	55500	61000	67000
O_H_	Position (eV)	532.6	532.6	532.6	532.6
	Area	7500	7000	7000	7500
O_M_/(O_M_ + O_V_ + O_H_) area ratio (%)		74.4	71.8	69.9	67.7
O_V_/(O_M_ + O_V_ + O_H_) area ratio (%)		22.4	25.0	27.0	29.1
O_H_/(O_M_ + O_V_ + O_H_) area ratio (%)		3.2	3.2	3.1	3.2

**Table 2:** The electrical properties of a-IGZO TFTs with various *T*_IGZO_.

Thickness (nm)	25	45	75	100
*μ*_sat_ (cm^2^∙V^−1^∙s^−1^)	14.17	14.01	13.62	13.04
*V*_on_ at *I*_DS_ = 1 nA (V)	2.32	0.27	0.04	−0.33
Hysteresis Δ*V*_H_ (V)	0.52	0.54	0.55	0.43
Subthreshold swing (mV/dec.)	323	367	416	475

**Table 3:** The statistical deviation of the electrical properties of a-IGZO TFTs with various *T*_IGZO_.

Thickness (nm)	25	45	75	100
*μ*_sat_ (cm^2^∙V^−1^∙s^−1^)	13.91 ± 0.92	13.70 ± 0.39	12.50 ± 1.01	11.88 ± 0.34
*V*_on_ at *I*_DS_ = 1 nA (V)	2.29 ± 0.28	1.60 ± 0.46	1.31 ± 0.44	0.88 ± 0.46
Hysteresis Δ*V*_H_ (V)	0.37 ± 0.18	0.33 ± 0.07	0.38 ± 0.27	0.28 ± 0.12
SS (mV/dec.)	279 ± 24	287 ± 19	314 ± 23	351 ± 26
